# Auditory scene analysis in music: A synthetic review

**DOI:** 10.3758/s13414-026-03310-y

**Published:** 2026-08-02

**Authors:** Kai Siedenburg, Robin Hake, Simon Jacobsen, Michel Bürgel

**Affiliations:** 1 Institute of Music, Carl von Ossietzky Universität Oldenburg, Oldenburg, Germany; 2 Department of Music, University of Leeds, Leeds, England; 3 Department of Medical Physics and Acoustics, Carl von Ossietzky Universität Oldenburg, Oldenburg, Germany

**Keywords:** Music perception, Musical scene analysis, Auditory scene analysis, Gestalt principles, Experimental paradigms, Multi-source music, Blend, Salience, Complexity, Inter-individual differences, Immersion

## Abstract

Music is the synthesis of a multitude of components: the combination of spectrotemporal structures emerging from different acoustic or electronic instruments, carefully orchestrated to form a connected whole. From these complex textures, our auditory system groups components through a process called musical scene analysis (MSA). In this review, we aim to address several questions about MSA. What are the perceptual principles underpinning musical scene perception? How are these principles being probed with different experimental paradigms? What stimulus and listener factors shape MSA? And what are future perspectives that further drive this field of research? We will find that Gestalt principles from psychology help us organize a musical scene through primitive and schema-based grouping cues. Such principles are being investigated through both minimalist and ecological experimental designs that give rise to several factors affecting MSA. We will present studies that have investigated blend and segregation, salience, and complexity, as stimulus-based aspects, and both peripheral and cognitive factors, which explain inter-individual differences among listeners. We conclude by exploring different perspectives on how music is attuned to MSA and the aesthetic affordances of multi-source music – perspectives that might inform future research in the field.

## Introduction

Music lives from synthesis – the combination of components to form a connected whole. It is the synthesis of stacked partial tones and their variation over time that builds the spectral structure of (in)harmonic complex tones and thus their identity. It is the multidimensional perceptual relation of complex tones and the way in which tones combine in frequency space that creates musical harmony. It is the synthesis of sounds from different acoustic or electronic instruments that yields the textures of ensemble (multi-source) music, whether mono-, poly-, homo-, or heterophonic (Huron, 2016), or textures that fall between or beyond these categories. Through synthesis on different scales of musical granularity, complex musical properties emerge while the perceptual identity of individual elements can be partially preserved (McAdams et al., 2022). Multi-source music does not simply add up as one musical entity, but usually retains parts of the identities of its individual components while creating new and interesting musical qualities. Consequently, multi-source musical textures by design invite listeners to perceptual analysis using the powers of the ear and the listening brain.

**Fig. 1:**
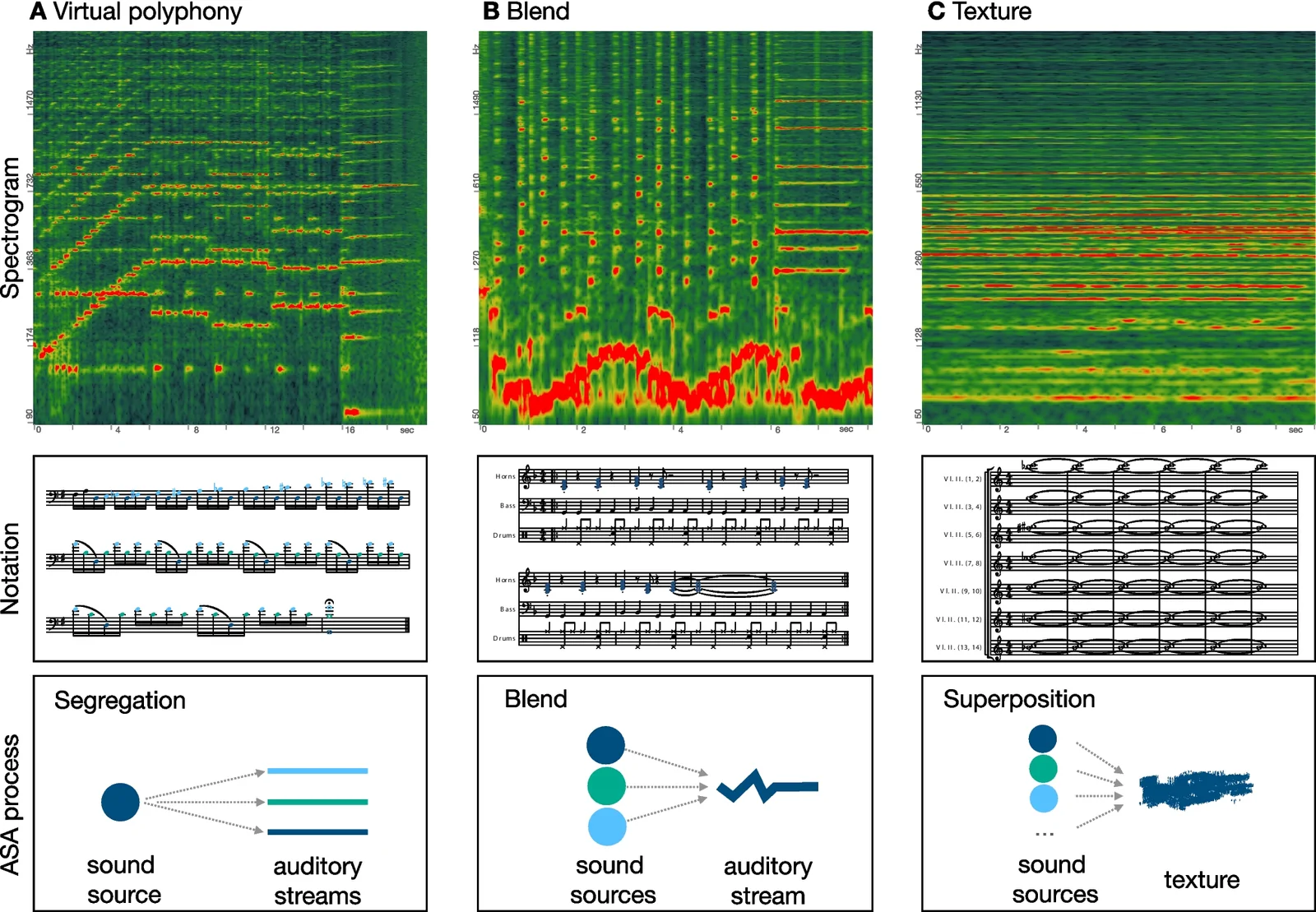
Musical scene analysis illustrated according to three examples. The *top panel* depicts a spectrogram of a musical excerpt, the *middle panel* its corresponding musical notation, and the *bottom panel* a schematic of the ASA process in action. **A** Pablo Casals playing a JS Bach cello suite (#1 in G major, BWV 1007) with three different streams emerging from one instrument. **B** An excerpt of the piece *Milestones* played by the Miles Davis jazz quintet, providing a fused brass section. **C** Ligeti’s *Atmosphere* played by the Berlin Philharmonics yielding a sound mass

This review synthesizes past and recent research on musical scene analysis (MSA) and attempts to make it accessible to (future) researchers in experimental (music) psychology. The term MSA derives from the parent category of auditory scene analysis (ASA), denoting the perceptual organization of sound in a noisy world, in which sounds from different sources tend to mix and overlap. ASA needs to parse the resulting mixtures to recover descriptions of individual sounds. Von Helmholtz (1885/1954) already distinguished different modes of listening to sound mixtures: whereas *synthetic* listening is geared towards the global aspects of sound, *analytic* listening focuses on details or local features. His prime example for synthetic listening was the perceptual impenetrability of the higher partials of a complex tone, which together support the perception of pitch. The prime example of analytic listening was hearing out individual low-numbered partials of complex tones (which arguably may require some degree of musical or auditory training). In the 20th century, various researchers started studying ASA around the same time (Bregman et al., 1971; Deutsch, 1975; van Noorden, 1975), now equipped with the toolkit of electronic sound analysis and synthesis. ASA was first applied to music by McAdams and Bregman (1979). Often considered a founding figure of ASA research, Albert Bregman pursued the most comprehensive research agenda in this area, leading to a theory outlined in his seminal book *Auditory scene analysis: The perceptual organization of sound* (Bregman, 1990).

Figure 1 presents three cases of MSA in action. Please note that sound examples corresponding to most of the figures of this manuscript are provided via a companion webpage^1^. Panel A shows a spectrogram from a recording of Pablo Casals playing a Bach cello sonata with three different streams emerging from one and the same instrument. This phenomenon is called *virtual polyphony*, because the polyphony is not veridically representing the physical world where only one sound source is present (the cello). By temporally interleaving distinct streams, this effect can also arise with truly monophonic instruments such as the flute, which is able to play only one tone at a time. Figure 1B depicts a case of blending in a recording of Miles Davis’s jazz quintet playing the beginning part of the tune *Milestones*. Here, sounds from the wind section (trumpet by Miles Davis, tenor saxophone by John Coltrane, alto saxophone by Julian “Cannonball” Adderley) blend together and thereby create a new (virtual) sound source of unique identity. Blend is among the core aims of orchestration and among the more intensely researched topics in MSA (see below). Figure 1C shows an excerpt of Gyorgi Ligeti’s *Athmosphères*, that results in a *sound mass*, a superposition of many musical lines in a string orchestra that each finely modulate in pitch and yet fuse together into one mass. As the composer puts it, “The polyphony in itself is almost imperceptible but its harmonic effect represents the intrinsic musical action: what is on the page is polyphony, but what is heard is harmony.”^2^ Such musical examples are hard to control experimentally in terms of their acoustical variables, but offer a window into “real-world” music perception. MSA research thus deals with a tradeoff between ecological validity and experimental control (a tension common to most other experimental research). In other words, the experimental literature on MSA spans a wide methodological range, from studies employing simple tones to investigations of full orchestral works. This range of approaches is reviewed in the following sections.

Among the most important and elementary terms in ASA is the auditory *stream*. As defined by Bregman, “An auditory stream is our perceptual grouping of the parts of the neural spectrogram that go together.” (Bregman, 1990, p. 9). The term stream thus is reserved for a perceptual representation, whereas the term *sound* refers to the acoustical event in the world; please see the Appendix for a glossary of central terms in MSA. Streams serve the purpose of clustering related qualities that extend over time, such as the tones of a melody. The mapping of sound sources to streams is not one-to-one: in some circumstances, sounds of one instrument can be perceived as multiple streams (see Fig. 1A), whereas in others, sounds from multiple instruments constitute one stream (Fig. 1B). Individual auditory events constitute parts of streams, and auditory attributes such as loudness, pitch, and timbre are mapped onto both events and streams (more on that later). This organization process is achieved through the extraction and evaluation of auditory cues via bottom-up and top-down processes. Bottom-up processes, often referred to as primitive or stimulus-driven processes, entail the extraction of sensory information from the acoustic input, analyzing the physical properties of sound signals such as fundamental frequency, onset and offset time, spatial location, or sound level (Bregman, 1990).

In contrast, top-down processes, also referred to as schema-based or knowledge-driven, involve the application of prior knowledge, expectations, intent, and selective attention to utilize contextual information and knowledge to interpret auditory scenes (Snyder et al., 2012). To complement the discussion of terminology, let us note that sound unfolds in time. Therefore, referring to auditory events and streams is more precise and does not assume a type of constancy over time that the term *auditory object* implies. Nevertheless, the notion of auditory object has been adopted by some researchers due to its conceptual appeal for domain-general accounts of perception that aim to draw parallels across sensory modalities, such as the face and voice of the same individual (Griffiths & Warren, 2004). A related notion of similarly general scope is the metaphor of the *auditory image*, a psychological representation of a sound entity exhibiting an internal coherence in its acoustic behavior (McAdams, 1984). The auditory image metaphor has some conceptual overlap with the notion of an auditory object.

From a psychoacoustic point of view, MSA is anything but trivial. More the rule than the exception in music, sound waves from multiple instruments overlap in time and frequency in musical mixtures. The resulting perceptual representations must be disentangled and mapped onto fused or segregated sound events to create structures apprehensible to the listener. How is this achieved? What are the underlying perceptual principles? What are key acoustical determinants and individual differences between listeners? How does the musical structure attune to MSA? In this review, we attempt to tackle these questions and provide an accessible introduction and springboard for future research on the topic. In the next section, we will provide an overview of canonical auditory Gestalt principles that equally apply to music and speech, before outlining the most important processes of stimulus-driven and schema-based grouping in music. This is followed by a taxonomy of experimental paradigms useful for characterizing research on MSA. We then offer an update on the latest empirical research on MSA that focuses on acoustical determinants of the music. A subsequent section focuses on recent research on individual differences between listeners. Finally, theoretical perspectives on MSA will be discussed to address the affordances of multi-source music more generally.

**Fig. 2:**
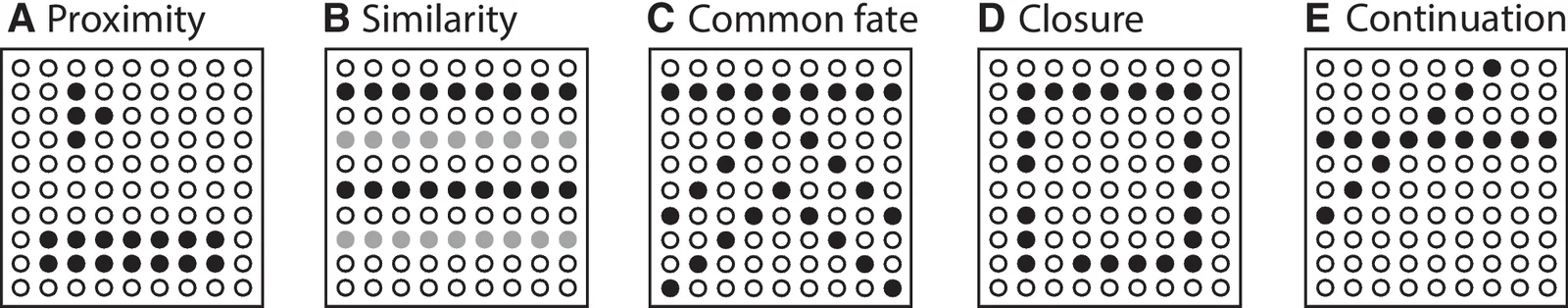
Visual illustration of Gestalt principles as foundation of auditory scene analysis. Depending on their arrangement, the *filled dots* (analogical to energy pixels in a spectrogram along time and frequency) form different groups. **A** Proximity: An upper group and a lower group are formed due to spatial (or spectrotemporal) proximity. **B** Similarity: *Black* and *grey lines* separate and form separate groups. **C** Common fate: Roof-shaped components form one group and contrast with the straight line on top. **D** Closure: a square is formed, despite missing information. **E** Good continuation: The trajectory of components is interpreted in the simplest form, yielding two crossing straight lines instead of two cornered lines

## Foundations of ASA

A commonly used analogy of ASA introduced by Bregman (1990) illustrates the fascinating capability of the auditory system. Imagine you sense the waves at the ends of two small canals connected to a lake. How much of the actions on the lake will you be able to infer? Can you tell whether a boat, surfboard, or swimmer was making a splash at a specific location in the lake? This is what the auditory system routinely manages with sound. Just as the canals provide only partial access to the dynamics of the lake, the ears receive only pressure fluctuations generated by sound sources at a distance. Nevertheless, the auditory system routinely solves this inverse problem with fascinating precision. In a concert, for instance, listeners are able to tell which instruments are making a splash (i.e., a sound) at specific locations on stage. The exact machinery of this system is hidden deep in the complex physiology and neural wiring of the auditory system. Research on ASA has approached this complex system by revealing simple functional principles, demonstrated under well-controlled conditions as minimal examples. Taken together, these grouping principles explain much of ASA even under more complex conditions, whether concerning speech perception at a cocktail party or music perception at a live concert.

### Auditory Gestalt principles

Research on auditory grouping was historically very much inspired by Gestalt psychology. The term *Gestalt*, derived from the German word meaning ‘shape’ or ‘form’, refers to the concept that the human mind tends to perceive objects holistically, integrating all their parts into a structured and meaningful pattern. In ASA, this implies that the auditory system extracts cues from auditory input and organizes them by (Gestalt) principles to determine whether they should be perceived as part of the same sound source (integration) or as separate entities (segregation). Sounds with cues that match or closely align are more likely to be integrated into an auditory stream, whereas those that differ are more likely to be separated into distinct streams. This organization is shaped by a set of auditory analogs to visual Gestalt principles. These principles include: *proximity, similarity, common fate, closure, and good continuation* (Deutsch, 2013b), illustrated schematically in Fig. 2.

As outlined in Fig. 2A, the principle of *proximity* involves grouping sounds that occur close together in frequency or time. For example, sound components close in time and frequency tend to fuse as one event. Tones presented in succession within a narrow pitch range likely form a single coherent stream. Figure 1A presents the example of virtual polyphony, where rapid alternation between high and low tones played on a single instrument can exceed proximity constraints and lead listeners to perceive two independent melodic streams.

Closely related is the principle of *similarity*, which posits that acoustic elements sharing comparable features are more likely to be grouped together. This is why a melody played by a solo instrument forms a single stream (and thus yields a melodic form), even when embedded in a dense orchestral texture. Likewise, multiple background vocal lines with closely matched timbre tend to fuse into a unified choral stream, despite the physical multiplicity of performers. Similarity is a critical feature in blending as discussed below.

*Common fate* refers to the grouping of sounds that change together over time. When two or more auditory elements exhibit synchronized fluctuations – such as simultaneous changes in frequency or amplitude modulation – they are perceived as being part of the same auditory source. Vibrato is a good example of how an instrumental or singing voice can stand out more clearly when frequency modulations alter its spectral properties coherently.

The principle of *closure* describes the perceptual filling-in of incomplete or interrupted sounds. Even when parts of a sound or melody are masked or missing, listeners often perceive the stream as continuous and intact. A saxophone line may momentarily be masked by a big-band hit or a strong drum accent, yet listeners still perceive its solo as continuing behind the interruption. Although the physical signal is briefly obscured, the auditory system maintains the melodic stream by “filling in” the missing segment.

The principle of *continuity* refers to the tendency to maintain a perceptual stream over time, even when the sound undergoes gradual changes or includes small deviations. A good example of continuity is the “scale illusion”, wherein two sources can play pitch sequences alternating between high and low, but the auditory system constructs two continuous sequences (Deutsch, 2013a).

**Fig. 3:**
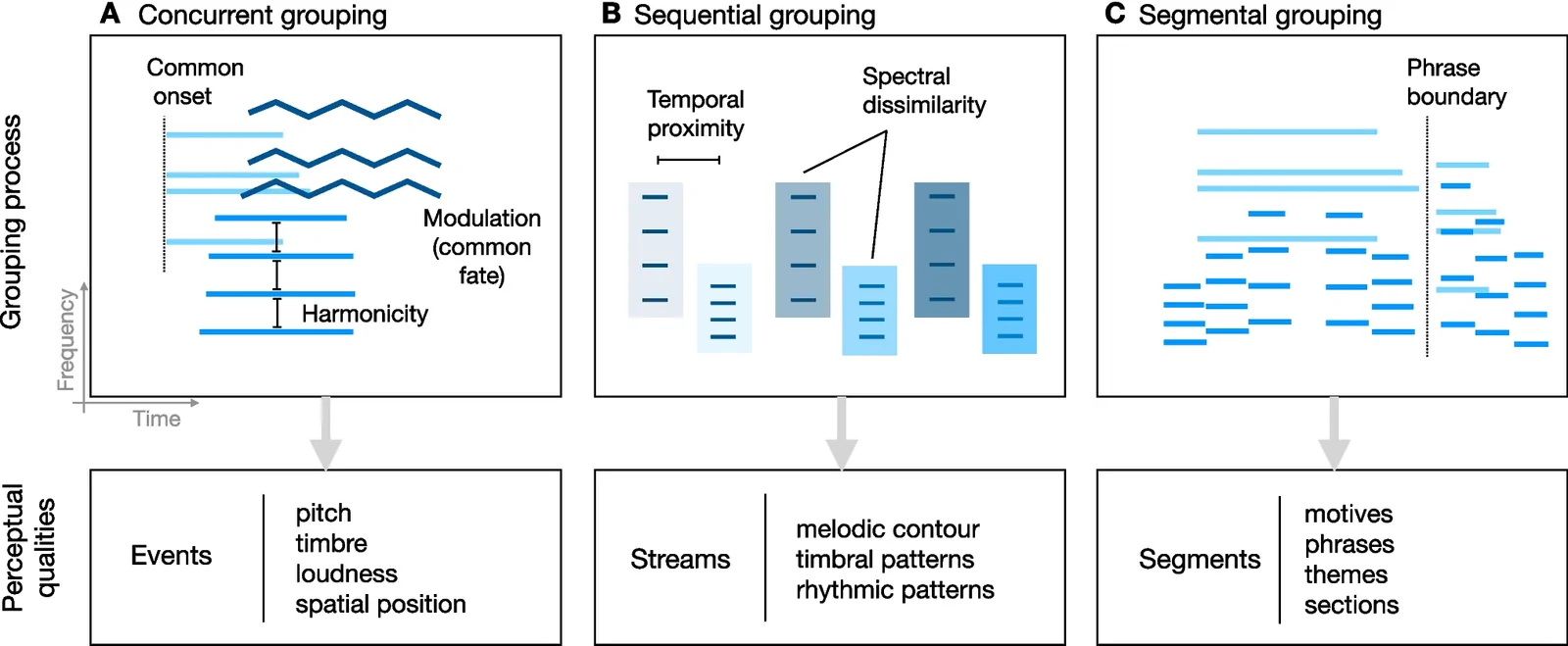
Fundamental aspects of auditory scene analysis: **A** concurrent grouping/simultaneous integration, **B** sequential grouping/stream segregation, and **C** segmental grouping. The *top panels* illustrate the process with a stylized time-frequency plot; the *bottom panels* list the resulting perceptual qualities that arise on the level of the auditory **A** event, **B** stream, and **C** segment

These canonical auditory Gestalt principles apply to music and speech equally and illustrate the parallels of perceptual organization between vision and hearing (Handel, 2019) . However, they pool various grouping processes and do not provide a systematic framework for differentiating stages of auditory grouping in musical scenes. This is the purpose of the subsequent two subsections on stimulus-driven and schema-based grouping processes.

### Stimulus-driven grouping in music

When it comes to stimulus-driven (also called *primitive*) grouping cues, three different stages in auditory grouping should be distinguished: concurrent (or simultaneous) grouping, sequential grouping, and segmental grouping (see McAdams, 2019). Even though these processes are thought of to be domain-general in audition, here we will focus on their role in music perception, where the output of these stages largely coincides with note-level events, phrases, and segments, respectively. Figure 3 illustrates these stages using stylized time-frequency plots.

At a first stage of grouping, the auditory system needs to determine which of the simultaneous components originate from common sources. This is the process of concurrent grouping (Fig. 3A). The *common onset* of acoustic components is among the strongest grouping cues. The transient sound of a piano hammer hitting a string has a very different spectrotemporal profile compared to the harmonic vibration emitted by the hit string (Siedenburg, 2019). Nonetheless, because the build-up of the string vibration is relatively rapid and its perceptual onset falls within a window of tolerance of a few tens of milliseconds, both components group together to form one auditory event. Notably, sounds with gradual onsets fuse better compared to sounds with sharp onsets (Bregman, 1990); note that there are valuable audio demonstrations accompanying the book that any interested ear should explore.^3^*Harmonicity* is equally critical as a grouping cue. The human voice, similar to most Western instruments, exhibits harmonically spaced partials. Pitch perception is based on the fact that harmonic partials are grouped together and heard as a single tone. Much research on pitch perception has revolved around the perceptual and neural mechanisms underlying this grouping process (Oxenham, 2012). Research based on the *mistuned harmonics* paradigm has shown that listeners tolerate 4% of frequency mistuning before a single isolated component “pops out” of a harmonic series (Moore et al., 1986). *Frequency (micro-)modulation* is yet another important cue that unites components with coherent behavior over time (McAdams, 1989). A prime example is vibrato, which lets singers and instrumentalists stand out much more clearly within a musical mix.

Overall, it is generally posited that event formation (fusion) precedes attribute formation. That is, concurrent grouping establishes basic auditory events that can be tagged with auditory attributes such as pitch, timbre, loudness, and spatial position (these are perceptual qualities in the minds of the listeners, not in the sounds themselves or the spectrogram). These perceptual attributes are intra-event properties, and do not extend beyond the unit of the event. As argued by Bregman (1990), “factors such as pitch, timbre, and location are the *results* of segregating the mixture, not the *causes* of its segregation.” (p. 531).

The second critical stage of MSA is to “connect the dots” of individual auditory events to form melodies and sequences, which is described as sequential grouping (Fig. 3B). The result of sequential grouping is the formation of auditory streams, that is, a mental representation of an event sequence with similar properties likely originating from one source. This grouping draws from similarity in event qualities (spectral, temporal, spatial) but also context information. Classic research using the *galloping horse paradigm*, two interleaved sequences of simple tones that create a galloping rhythm (see section on Experimental paradigms below), has shown that the inter-onset time of tones and the frequency proximity of the two sequences determine whether they are heard as one or two melodies, but that there is a region of ambiguity, where listeners may switch (van Noorden, 1975). Musical patterns, whether melodic, timbral, or rhythmic, arise within auditory streams, not across. For example, it is hard to process detailed rhythmic information across perceptual streams. Stream segregation also underpins the formation of musical structures in terms of a foreground, middle ground, and background, and is thus at the heart of orchestration (McAdams et al., 2022). Concerning the relation between concurrent and sequential grouping, one should note that these two processes are not operating in an entirely independent manner. When pitting concurrent and sequential grouping against each other, early demonstrations by Bregman et al. (1978) suggested that sequential grouping operates on the output of concurrent grouping, as well as on context information in the sound sequence.

Finally, segmental grouping (Deliège, 1989) concerns the formation of oftentimes hierarchical musical structures (see Fig. 3C). That is, motives, phrases, themes, and section boundaries are computed by segmental grouping processes. In orchestral music, the evoked contrasts are often based on timbre (McAdams et al., 2022).

In recent research, machine learning models were trained on tasks related to ASA to derive principles from the learned representations (Cusimano, 2022; Młynarski & McDermott, 2019). The success of these approaches suggests that grouping principles are not some mysterious properties of the mind, but are heuristics extracted from the regularities of the acoustical environment that help to group sources with common causes.

### Schema-based grouping in music

A *schema* is an abstract knowledge structure shared by different occurrences of the same type of musical sounds and sequences (e.g., think of the regularities of tonal music). Besides a primitive, bottom-up segregation mechanism that may essentially be considered as source separation, researchers have argued for a second mechanism that draws from prior experience and mental schemata. Bregman (1990) proposed the notion of *schema-based* segregation that lumps together contributions from attention and memory and is assumed to be less automatic and more akin to a process of information selection. In other words, whereas primitive segregation offers separated “tracks of the neural spectrogram”, schema-based segregation selects “tracks” of interest and discards the rest.

This schema-based mechanism falls on fertile ground for music, which, as an acoustic stimulus, is highly regular and redundant (Margulis, 2014). Music perception is attuned to such regularities. As an example, consider how tonal hierarchies constrain pitch expectations, how metrical frameworks shape rhythmic perception, or how the properties of musical instruments shape idiomatic performance patterns. Thus, it is highly plausible that MSA also uses knowledge about stylistic regularities of music, rather than purely stimulus-driven cues. However, empirical studies on schema-based contributions to segregation are still relatively scarce.

An important piece of evidence for the importance of memory and attention comes from a study by Bey and McAdams (2002). They had listeners recognize interleaved melodies and had them judge whether the target melody was identical to a melody in the mixture or not. They found that presenting a target melody before the mixture (so that listeners knew what to listen for) yielded much higher segregation compared to presenting the target after the mixture, supporting the role of attention in stream segregation. McDermott et al. (2011) showed that repeating noise-like target signals are better segregated from competing signals compared to non-repeating target signals. Similarly, Woods and McDermott (2018) suggested that auditory schemata are learned and employed in scene analysis tasks. In their experiments, a subset of trials contained schema-based sources generated from a template by transformations, introducing acoustic variation but preserving abstract structure. Notably, across several tasks and classes of sound sources, schema-based sources consistently aided source separation. Similar to the rapid implicit auditory memory of noise tokens (Agus et al., 2010), in some cases the presence of schemata yielded rapid improvements in performance over the first few exposures.

In natural music, accompaniments especially tend to be highly repetitive. It seems plausible that this contributes to listeners’ tendency to focus on and follow the melodic lines. Taher et al. (2016) studied attention in two-part counterpoint textures. Participants were presented with musical excerpts that contained a repetitive and a nonrepetitive part, and provided ratings of the relative prominence of the two voices. The authors observed that the line that consists of immediate and exact repetitions tended to perceptually decrease in salience for the listener.

The implications of these studies for real-world musical scenes seem clear: listeners build an internal model of the musical scene that facilitates grouping. Such model building appears to be particularly straightforward for familiar musical genres, where not only the repetitiveness of the music but also long-term knowledge of the musical structure aids this process.

## Experimental paradigms

Various experimental paradigms have been used to characterize MSA. A general distinction may be drawn between appearance and performance tasks (Kingdom & Prins, 2016). In appearance tasks, subjects provide an assessment of the appearance of stimuli (e.g., “I hear two streams.”). Or in the words of Bregman (2015, p. 12), “Since I have always wanted to find out what listeners were actually hearing, I have taken the liberty of asking them.” Appearance tasks do not have a right or wrong answer or an objective ground truth, but tend to be very efficient, because every trial yields one numerically graded response. Performance tasks, on the contrary, have a ground truth in the form of correct or wrong responses, but only indirectly measure aspects of scene perception. For instance, one may measure melodic discrimination in the presence of an interfering signal and one can indirectly infer that the cause of higher performance lies in more stable stream segregation of the target melody. Both types of tasks provide important information about MSA. Research on stream segregation has found that listeners do not seem to bias their perceptual reports due to possible implicit expectations present in the experimental context (Farkas et al., 2016), which underlines that valid perceptual data can be obtained from both appearance and performance tasks.

A second distinction regarding experimental paradigms can be made with respect to their complexity. Paradigms in ASA research live on a continuum from simple/controlled/musically unrealistic to complex/not completely controlled/musically more realistic. Here, we refer to the former as *minimalist designs* (sometimes also referred to as atomistic), which may be considered as analogical to minimal examples in programming, suited to demonstrate a certain function (or bug) under the simplest conditions. In the case of audition, such designs are suited to demonstrate a certain perceptual process and to rule out confounding factors. The latter may be computer-generated stimuli or excerpts from records, selected for the purpose of ecologically valid experimentation. Ecological designs are especially suited if interest lies in the effects that specific musical structures have as a whole. Often, the interpretation of results and potential acoustical factors is constrained by using acoustical stimulus models. Even though researchers vary in their preferences for one or the other, both are important parts of the experimental toolkit needed to obtain a comprehensive understanding of MSA.

### Minimalist designs

**Fig. 4:**
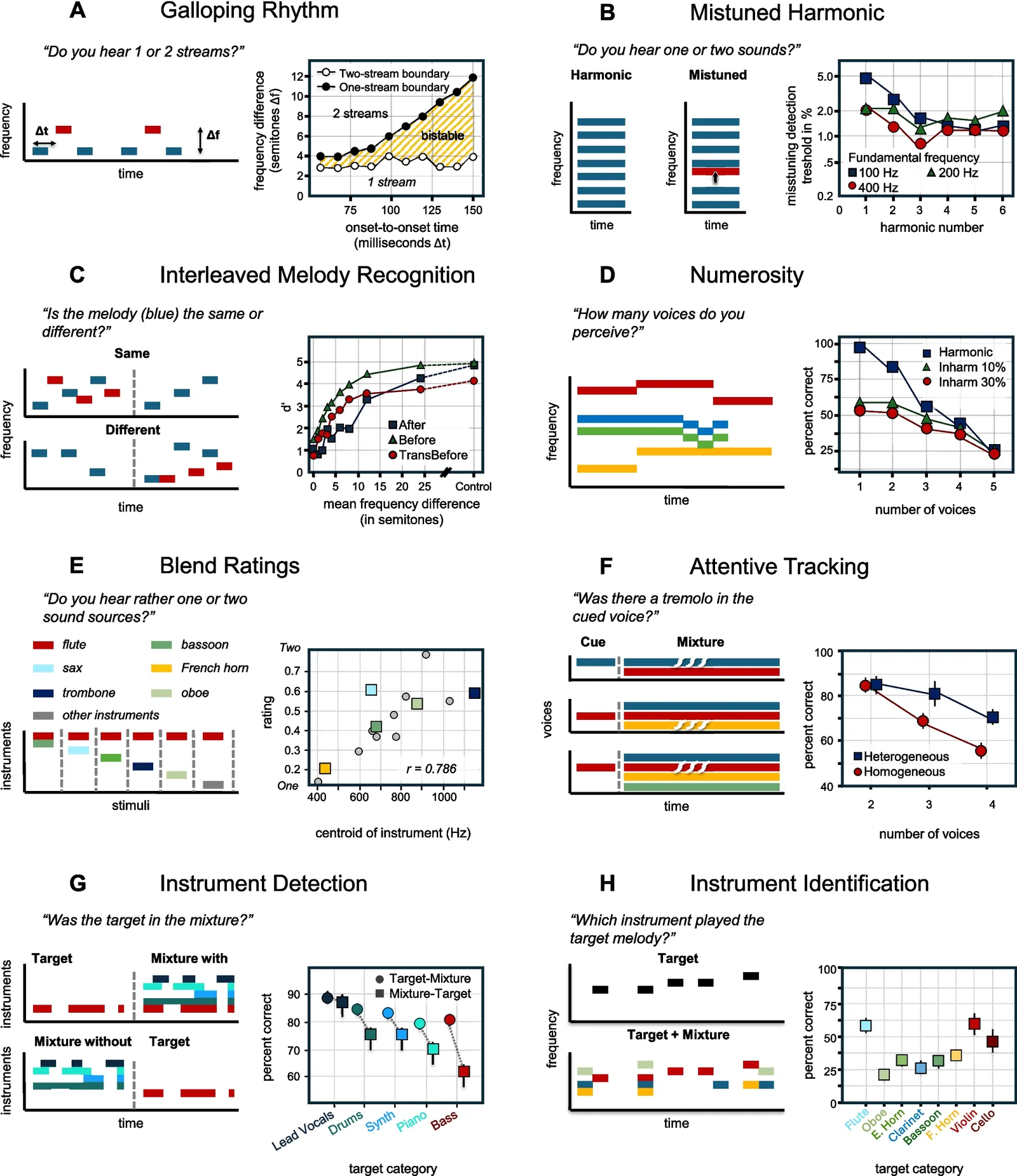
Experimental paradigms in MSA: Schematic of methods (*left*) and representative results (*right*). **A** Galloping Rhythm (van Noorden, 1975), **B** Mistuned Harmonic (Moore et al., 1986), **C** Interleaved Melody Recognition (Bey & McAdams, 2002), **D** Numerosity (Bogaard et al., 2025), **E** Blend Ratings (Sandell, 1995) (*gray dots * represent a variety of other instruments of the study which are not specified in the legend), **F** Attentive Tracking (Siedenburg et al., 2021), **G** Instrument Detection (Bürgel et al., 2021), **H** Instrument Identification (Jacobsen et al., 2026)

A seminal contribution by van Noorden (1975) introduced a paradigm designed to probe how temporal and spectral proximity interact in stream formation. Listeners were asked whether they heard one or two streams (appearance task). As illustrated in Fig. 4A (and in the accompanying online interactive sound examples linked above), the stimulus consisted of a repeating ABA triplet: two identical low-frequency tones (A) surrounding a higher-frequency tone (B), producing a characteristic “galloping” rhythm, and illustrating stream formation in one of the simplest ways possible. When the A and B tones are close in frequency and time, listeners perceive them as one unified stream with a galloping rhythm. As the frequency separation between A and B increases, the perceptual organization shifts, resulting in stream fission, and two separate streams are heard, B at one tempo and A at twice that tempo. Temporal spacing also plays a critical role, as decreasing the inter-tone interval promotes stream segregation. Notably, the spectral and temporal boundaries are interdependent: faster presentation rates reduce the frequency difference required for segregation. Moreover, within an intermediate range, perception may alternate between integrated and segregated interpretations or may vary strongly between individuals, reflecting the bistable nature of auditory streaming. This paradigm has been used in a large number of studies over the years and embodies the most canonical illustration of the effects of sequential stream formation.

Principles of concurrent grouping were examined in a classic paradigm by Moore et al. (1986). Here, the degree of mistuning required for a harmonic component within a harmonic tone complex to be perceived as a separate tone was tested. An outline of the methods and results is displayed in Fig. 4B. Participants listened to harmonic tone complexes composed of 10–12 partials. One of the first six components was mistuned (shifted slightly up or down in frequency) and listeners responded whether they heard one harmonic complex or a complex plus a separate tone. An adaptive procedure was used to estimate the mistuning threshold at which the altered tone popped out and was no longer perceived as part of the harmonic complex. Thresholds typically ranged around 2% of the respective harmonic frequency for sounds of 410-ms duration, but results also depended on the harmonic number of the mistuned partial tone. This by now classic paradigm has since then served as a popular minimal example to demonstrate aspects of concurrent grouping and pitch perception. The paradigm also reveals interesting insights into auditory attribute formation (computation of pitch, timbre, loudness, etc.). Measuring the effects of mistuning on the perceived pitch of the harmonic complex (regardless of whether the mistuned harmonic was heard as separate or not) revealed that even though listeners tended to hear two sound events at magnitudes of around 2% mistuning (Moore et al., 1986), increasing mistuning up to 4% altered the pitch of the harmonic complex (Moore et al., 1985). That is, the computation of pitch can be influenced by a segregated auditory element, challenging the notion raised by Bregman (1990), who argued that attributes are computed on grouped components after concurrent grouping has taken place. Concretely, attribute formation may partially rely on pre-grouped representations and both processes may indeed have some “leakage”.

A more cognitively driven approach to testing ASA boundaries was introduced by Dowling (1973) and elaborated by Bey and McAdams (2002), utilizing a paradigm to probe schema-based and attentional influences on auditory grouping. An outline of the methods and key results is displayed in Fig. 4C. Participants were asked to judge whether two sequentially presented pure-tone melodies were identical. One of the melodies was interleaved with a distractor melody, making the comparison task more demanding. In the first condition (“After”), the interleaved melody is presented first, followed by the same melody in isolation. In the second condition (“Before”), this order is reversed: an isolated melody was presented first, allowing listeners to form a mental template before encountering the interleaved version. This manipulation tests in a straightforward way whether prior exposure to a structured auditory pattern guides selective attention and facilitates stream segregation. A third condition (“TransBefore”) introduced an additional challenge. Although the isolated melody was again presented first, it was transposed to a different frequency range relative to the subsequently presented interleaved melody. This condition demonstrated that relative pitch relationships support attentional guidance. Bey and McAdams (2002) further explored acoustic influences by varying the frequency distance between the target melody and the distractor, ranging from 0 to 24 semitones. Here, two main effects emerged. First, increasing spectral separation between the target and distractor improved performance, confirming the role of frequency distance in stream segregation. Second, performance was significantly enhanced when the isolated melody was presented first – even in the transposed condition – demonstrating that cognitive factors such as expectations and attentional priming can support ASA. As opposed to the appearance-based “galloping rhythm” task, interleaved melody recognition constitutes a flexible performance-based task of sequential stream formation that has been re-used many times (e.g., Woods and McDermott, 2018).

### Ecological designs

Real-world auditory scenes often consist of multiple overlapping and dynamically varying sound sources. A way to examine the limits of ASA in such scenarios is to test how many concurrent sound sources listeners can distinguish in a scene. This was tested by Huron (1989), who explored the perceptual limits of polyphonic music. In the experiment, listeners were presented with musical excerpts containing up to five simultaneously playing melodies that entered, left, and reentered the musical scene. Relatively homogeneous-timbre instruments played the melodies. Performance declined markedly beyond three voices. Listeners were also more successful at detecting the start and end of the highest and lowest melodic lines, while melodies in between were more likely to be confused or missed. Huron (1989) concluded, perhaps with a touch of sarcasm, that auditory perception effectively counts “one, two, three, many” (p. 19). Converging evidence was reported by Bogaard et al. (2025), who collected numerosity judgments in an investigation of the role of harmonicity. Participants were asked to judge the number of concurrently singing voices with inharmonicity introduced by randomly jittering harmonic components of tones by ±30% of their respective fundamental frequency. An outline of the methods and results is displayed in Fig. 4D. The results demonstrated that increasing inharmonicity impaired numerosity judgments in a manner comparable to adding additional sound sources. Although numerosity judgments provide valuable insights into scene perception, it remains questionable, however, whether they are a sharp measure of MSA, because working memory restrictions yield an upper bound to the number of reliably reported sources. This also holds for speech scenes, where Kawashima and Sato (2015) found that listeners systematically underestimated the number of concurrent voices when their number exceeded three.

Sandell (1995) investigated instrumental blend by presenting pairs of musical instruments matched in onset, duration, and fundamental frequency, and asking listeners to judge whether the combination was perceived as one or two timbres (appearance task). Figure 4E provides an overview of the experimental design and results for the example of a flute sound, paired with sounds from the bassoon, saxophone, French horn, trombone, oboe, and various other orchestral instruments (the latter are lumped together and represented by gray dots for the sake of simplicity). The advantage of direct blend ratings is their experimental efficiency, the graded response, and the ease of implementation. In the study by Sandell (1995), perceptual blend was dominated by relationships of the spectral centroid (the center of gravity of the sound spectrum, corresponding to sound brightness) and the spectral centroid of the overall dyad. Here, similarity of the centroids of the paired sounds promoted fusion, whereas centroid disparity facilitated segregation, regardless of instrument identity. Temporal coherence cues – such as attack similarity, level change, and offset synchrony – modulated blend in a context-dependent manner, with different cue weightings for unison and non-unison pitch relations. A number of subsequent studies have used direct ratings to study blend and found stable and interpretable results (see section on Blend and segregation below).

While perceptual blend highlights conditions under which bottom-up grouping fails due to excessive similarity between sound sources, results from the interleaved melody paradigm demonstrate that top-down attentional processes can actively guide auditory grouping. In particular, prior exposure to a target pattern can bias attention and facilitate segregation, suggesting that attentional mechanisms may compensate for limitations of purely acoustic cues. Building on these ideas, Woods and McDermott (2015) investigated how attention operates when sound sources are not only similar, but also dynamically evolving over time, such that momentary feature overlap and blending are likely to occur. Using synthetic voices that varied continuously in pitch and timbre along intersecting trajectories, the authors created stimuli in which no source possessed stable distinguishing features. Listeners were cued to attend to one voice and later judged whether a terminal segment (probe) belonged to the attended source. Listeners were able to perform this task by attentively tracking the target as it moved through the feature space, rather than relying on any consistent distinguishing features. Yet performance broke down when source trajectories passed too closely, and thus did not maintain sufficient separation. These findings suggest that a movable locus of attention can support source continuity over time and mitigate failures of segregation, thereby complementing bottom-up grouping cues in dynamically changing auditory scenes. Extending this principle from synthetic stimuli to musical contexts, Siedenburg et al. (2021) adapted the paradigm to polyphonic music using excerpts from J. S. Bach’s *Art of the Fugue* by tasking participants with judging whether a cued target contained a tremolo or not. An outline of the methods and results is displayed in Fig. 4F. Their results showed that tracking performance declined with increasing number of voices, but that this effect was attenuated when voices within a mixture were timbrally heterogeneous. The tracking paradigm indexes an important aspect of auditory grouping, but it tends to be demanding for participants. In Siedenburg et al. (2021), only participants with a solid musical background were recruited for that reason. And even the original paradigm tends to be too difficult for many naive participants (cf., Madsen et al., 2019).

Extending the interleaved melody paradigm to an instrument detection paradigm, Bürgel et al. (2021) used real-world musical mixtures as stimuli. Rather than using artificial tone sequences, participants were presented with short musical excerpts derived from multitrack recordings. An isolated instrumental track is employed as the target, while the mixture of remaining tracks functions as the distractor context. Target and mixture are played with a pause in between. Participants are tasked to judge whether the target was present in the mixture or not. As in the interleaved melody paradigm, presentation order can be manipulated to assess attentional priming effects. An overview of the experimental design and results is provided in Fig. 4G. In this paradigm, stimuli can be directly generated from music, given that individual instrumental tracks are available.

Results revealed significant effects of both target instrument category and presentation order, as well as interaction effects between the two. In line with findings from the interleaved melody paradigm, presenting the isolated target instrument before the mixture facilitated detection, highlighting the role of attentional priming. Notably, lead vocals showed robust detection performance regardless of presentation order, whereas all other instrument categories were negatively affected when the isolated target was presented after the mixture. This decline was most pronounced for the bass instrument; follow-up studies using the same perceptual task but more tightly controlled stimuli suggest that this particular drop for bass instruments is likely due to musical factors, rather than processing biases regarding absolute frequency ranges (Bürgel et al., 2024). Generally, the paradigm yields a flexible tool to probe MSA under realistic conditions. At the same time, this naturally yields many potential confounding variables to be ruled out via acoustic modeling or additional experimentation.

Beyond detection tasks, MSA can also be investigated through identification paradigms, which address not only whether a sound was perceived, but also what kind of sound category was perceived. Identification tasks encompass a variety of approaches, ranging from recognizing which sound sources are present in an acoustic scene to speech perception paradigms that require listeners to identify individual speakers or their utterances. In music, a close parallel can be drawn by asking what content was played and which instrument within the mixture played it. Identification paradigms probe not only whether a sound can be identified, but under which conditions its identity breaks down. Such paradigms may include identification under masking, transformations across pitch or register, systematic degradation or removal of acoustic cues by modifying sound characteristics (e.g., fundamental frequency or partials), as well as manipulations of how sounds are presented (e.g., degree of spatial separation). An ecologically motivated identification paradigm was utilized by Jacobsen et al. (2026), who investigated how instrument identification in musical mixtures varies across instrument classes and acoustic listening conditions. The study employed a cue–mixture identification task using short excerpts from Richard Wagner’s Tristan prelude as source material. Participants were first presented with a cue melody consisting of pure tones and were then asked to identify which of the eight possible instruments played the cued melody within a subsequent excerpt. Each excerpt consisted of a mixture of four simultaneously playing instruments. A schematic overview of the experimental design and main results is shown in Fig. 4H. Results revealed instrument-dependent effects: identification performance varied systematically with instrument class, voice position within the mixture, register, and acoustic similarity between target and non-target instruments. This specific task has its benefits, such as a low-chance baseline determined by a relatively high number of response alternatives, but it is also a very demanding task. Listeners need to have a robust perceptual template of all target instruments in addition to the ability to hear out the target pitch from the mixture, which renders it difficult for many participants.

## Stimulus-based determinants of musical scene analysis

MSA happens in the mind of the listener, but the musical stimulus clearly drives this process. Now that we have given an overview of the basic experimental toolkit of MSA research, here we review three important phenomena in MSA that concern how the organization of musical mixtures is determined by the blending of sound sources, by their salience, and by their complexity. All of these topics are the focus of ongoing research.

### Blend and segregation

A central aspect of ASA is the separation of sound sources and their identification. Even if sounds are presented concurrently, ASA principles allow the auditory system to distinguish them from each other and attribute them to separate sources. However, this ability can be impaired due to the nature and presentation of the individual sounds. In music, when dealing with musical instruments as sound sources, this inability to separate them is often purposefully evoked by composers by choosing different combinations of instruments and playing techniques. The result is a blend of the constituent instruments in a sound mixture.

Blend describes the perceptual fusion of two or more concurrent sound events into a single fused entity. This fusion heavily depends on the sounds’ timbre. First scientific investigations into the fusion of different tones and their perceived timbre date back to Von Helmholtz (1885/1954). In *On the Sensations of Tone*, Helmholtz describes the fusion of tones from the blowing of two glass bottles pitched an octave apart, in which two tones become a single entity. Although this fusion of the same “instrument” primarily describes the perception of partial tones, it is an early report on blend. With respect to different musical instruments, Sandell (1995) conducted one of the first thorough investigations on blend. Synthesized instrument sounds were used to examine the factors that contribute to instrument blend in dyads at unison or a minor third apart. The results showed that blend ratings increased for instrument couplings with similar attack envelopes and spectral centroids, and for lower composite spectral centroids (i.e., of the mixture signal). Kendall and Carterette (1993) obtained converging results on spectrotemporal similarities. Both studies were concerned with pairs of sustained instruments. Tardieu and McAdams (2012) investigated combinations of pitched impulsive and sustained sounds. They found that the perceived blend of the dyad is governed by the attack of the impulsive sound and the spectral envelope of the sustained sound. More specifically, blend ratings increased with longer attack times and lower spectral centroids of the sustained sound. Besides simple acoustic descriptors of timbre, more complex aspects like formant structures also inform the levels of blend, as investigated in wind and brass instruments by Reuter (2002). Such formants describe prominent maxima in instruments’ spectra and are mostly invariant to changes in pitch. Lembke and McAdams (2015) further found that changes in formant center frequencies and formant prominence affect blend when parametrically varying a synthesized sound in a dyad with a recorded sound.

Whether or how well instrument sounds blend does not only depend on the spectrotemporal characteristics of the sounds themselves (source-level blending), but also on the room-acoustical characteristics of the space in which they are played. Thilakan et al. (2025) investigated the influence of room acoustics on the source-level blending between two violins. They reported source-level blending to contribute 60% and room acoustics 40% to perceived blend ratings. More concretely, enhanced reverberation and spatial envelopment increase the perceived level of blend, especially if initial source-level blending is poor.

So far, these studies have focused on controlled stimuli consisting primarily of concurrent instrument dyads. But how does blend play out in real musical excerpts? McAdams et al. (2025) investigated several factors that contribute to instrumental blends in orchestral excerpts. The authors collected perceived blend ratings for 64 excerpts, predominantly from the Romantic era. They found that timbre class (instrument family) strongly affected the ratings, with the highest perceived blend ratings for sustained strings, woodwinds, and their combinations. Combining impulsive sounds with sustained instruments yielded the lowest blend ratings. Furthermore, parallel pitch motion and onset synchrony led to higher blend ratings, both a result of concurrent grouping. Spectral factors related to timbre also affected blend ratings, with reduced spectral density and less spectral variation resulting in higher blend ratings.

Blend is a result of concurrent grouping, but it can also inform our perception of sequential grouping. Fischer et al. (2021) found that heterogeneous instrument combinations resulted in perceptually more segregated streams compared to homogeneous combinations. Although blend ratings on the individual streams did not globally predict stream segregation, a decrease in timbral differences of the constituent instruments yielded reduced segregation. In a recent study, Fischer and McAdams (2025) extended their findings to perceptual layers in orchestral music in the context of foreground and background. This shows how composers use different orchestration approaches to shape our perception of the music. But the written orchestration alone is not the only factor that facilitates blend by default. How well two instruments blend is also performance-based and depends on the players’ coordination. Lembke et al. (2017) investigated the blending between the horn and bassoon during musical performance. In a leader–follower setting, one of the players (follower) had to react to the other player (leader) and create a blended sound. Acoustical analyses of the performances showed a darkened use of timbre (lower spectral centroid) accompanied by a reduced dynamic level in the follower’s sound. Testing leader–follower relations in a live, improvised concert of a clarinet duo, Canonne et al. (2026) found that listeners’ continuous self-reports of selective attention tracked the performance instruction of the musicians. Specifically, listeners more likely focused on the musician who acted as a temporal leader in the improvisation and shifted attention more frequently when players used more contrastive material. Schwarz et al. (2025) even observed changes in the attentional state of improvising musicians due to the salience of the musical material. Studies of this type seem particularly suited to map out the complex dynamics of auditory attention in diverse musical settings.

A different approach to blend and attention than self-report measures is an instrument identification task. The interplay between identification and blend was already addressed by Sandell (1995) as they propose three types of blends: *timbral heterogeneity*, where the constituent instruments are not fused and remain identifiable, *timbral augmentation*, where one instrument sound is emphasized by another resulting in partial identification of the emphasized instrument, and *timbral emergence*, where a new timbre is created but the instruments are unidentifiable. Overall, this means that there is an inverse relationship between the subjective rating of blend and objective accuracy in identification, which has already been observed by Kendall and Carterette (1993).

A recent study (Jacobsen et al., 2026) explored instrument identification in a realistic acoustic context. Relationships between identification performance and the acoustic similarities of target and mixture instruments were found, as well as instrument-dependent effects of voice position within mixtures and instruments’ registers. Contrary to the findings on source-level blending (Thilakan et al., 2025), the study found only marginal effects of room acoustics on participants’ identification performance, although sound quality judgments were clearly affected by reverberation and the distribution of sound sources. Critically, even collapsing all sound sources to one location on stage did not exert a strong detrimental effect on identification performance, questioning the role of spatial cues in supporting segregation and identification in realistic musical scenes, and provoking future research on the issue.

In sum, blend is a perceptual result of concurrent grouping, wherein two or more instrument sounds are grouped together and can be used as a tool in orchestration. The perceived level of blend depends on spectrotemporal (timbral) features, room-acoustic parameters, the orchestration, and player performance.

### Salience

In musical mixtures, certain sounds are particularly effective at capturing auditory attention, a form of perceptual prominence known as auditory salience. Salient sounds possess characteristics that can involuntarily draw a listener’s attention, irrespective of their current attentional focus (Kothinti & Elhilali, 2023). Salient properties emerge from a combination of bottom-up acoustic cues, such as an instrument playing at a relatively high sound level, and top-down cortical processes, such as the violation of expectations or musical conventions through an out-of-tune note (Huang & Elhilali, 2017). The influence of these cues can shift as the musical mixture evolves over time and may also be modulated by the focus of auditory attention (Alain & Woods, 1997).

Various sources of salience within the compositional structure of musical mixtures have been identified, particularly when multiple melodies are played simultaneously. As a musical scene evolves, new melodic lines tend to draw attention, while repeated lines lose their perceptual prominence (Taher et al., 2016). Similarly, when multiple rhythmic motifs are presented together, those with irregular patterns are more likely to capture the listener’s attention (Jones et al., 1981), especially when attention is preemptively focused on specific motifs (Devergie et al., 2010). In musical pieces with a leader–follower hierarchy, the main melody or leader is typically perceived as more salient than the accompanying melodies (Ragert et al., 2014).

In two-voice polyphonic music, the melody with the highest pitch trajectory generally attracts more attention than the lower one – a phenomenon characterized by Fujioka et al. (2005) as the “high voice superiority effect”. Trainor et al. (2014) showed that this effect may arise from the perceptual suppression of harmonic structures, where the harmonic elements of the lower melody are masked by those of the higher melody. Evidence supporting this theory includes the presence of this effect in infants (Marie & Trainor, 1991), indicating that it is rooted in fundamental principles of auditory perception rather than learned musical conventions. Moreover, this effect occurs in musicians regardless of their instrument, though it was observed to be more pronounced in those who play soprano-range instruments compared to those who play bass-range instruments (Marie et al., 2012). Conversely, the onsets within the lower pitch trajectory act as particularly salient cues for beat and rhythmic perception (Hove et al., 2014). Collectively, these findings demonstrate that spectral and temporal aspects of music perception can be shaped by competing cues. This duality aligns with the common musical practice where the lead melody is typically played by higher-pitched instruments, naturally drawing attention, while the rhythmic foundation is usually provided by bass instruments. When examining mixtures with more than two musical voices, salience tends to be strongest for melodies at the outer edges of the musical mixture, thus the lowest and highest occurring melodies (Bürgel et al., 2024). There seems to be much room for future research to more comprehensively model the acoustic and auditory features that conjointly yield high voice superiority in diverse musical contexts.

Another cue that contributes to salience in musical mixtures is timbre. Chon (2013) hypothesized that each timbre possesses a unique degree of timbral salience, which is highly dependent on the timbral space it occupies within a musical scene. This concept remains relatively unexplored, as most studies on timbre and salience have focused on mixtures of environmental sounds rather than musical mixtures (Huang & Elhilali, 2017; Kaya et al., 2020). Recent work on orchestral listening, however, demonstrates that timbral contrast plays a central role in shaping the perceptual prominence of foreground layers in musical scenes (Fischer & McAdams, 2025). In particular, layers composed of instruments from different families exhibit greater perceptual prominence than overlapping or homogeneous combinations, highlighting timbral contrast as a key contributor to prominence.

Interestingly, several studies suggest that the human singing voice (i.e., vocals) stands out as a particularly salient element in auditory perception. In a study by Bürgel et al. (2021), the salience of various instrumental and vocal sounds in mixtures of popular music was investigated. The results highlighted differences between the sounds tested, with vocal sounds exhibiting a unique level of salience that surpassed that of all instruments, even when the sounds were matched in terms of level or filtering. Evidence supporting such vocal salience was also reported in neurophysiological studies, which not only demonstrated that speech is capable of activating specialized regions in the human cortex (Belin et al., 2000) but also elicits unique voice-specific responses to the exposure of singing voices, distinct from responses to instrumental sounds (Levy et al., 2001).

In subsequent experiments investigating the acoustic origins of vocal salience, Bürgel and Siedenburg (2023) demonstrated that the presence and intensity of frequency micro-modulations inherent in the singing voice significantly contribute to its salience. Moreover, eliminating these modulations was found to diminish the salience of singing voices. These micro-modulations partly arise from the imperfect pitch intonation typical of human singing (Hutchins et al., 2014) but can also be emphasized to enhance emotional expressiveness (Larrouy-Maestri et al., 2024; Sundberg et al., 2013). Importantly, vocal salience should not be understood as an unconditional privilege, but as a context-dependent phenomenon. Recent work on the recognition of short vocal and instrumental sounds in the presence of a piano masker showed that vocals exhibited heightened salience only when they were spectrally distinct from competing instruments (Bürgel & Siedenburg, 2024). This finding underscores that vocal salience depends on relative timbral contrast, aligning with evidence that prominence emerges from acoustic dissimilarities in the competitive interactions between timbres (Fischer & McAdams, 2025).

### Complexity

Polyphonic music compositions can certainly be complex, but how much of that complexity are listeners aware of at any given moment? Some authors have argued, especially with regards to the high number of concurrently active sound sources in music, that “music [...] arguably provides some of the most complex acoustic scenes that a human listener will ever encounter” (Pressnitzer et al., 2011). On the other hand, music is made for listening by design and musicians (may mostly be assumed to) make their music intelligible to listeners. According to Huron (2001), the *limited density principle* states that the tracking of voices becomes challenging when the number of simultaneously active voices exceeds three. Empirical studies with numerosity-judgment tasks have supported this idea. Experiments using polyphonic excerpts from the music of JS Bach indicated that errors in both numerosity judgments and the recognition of single-voice entries sharply increased from around ten percent for three-voice mixtures to around 50 percent for four-voice mixtures (Huron, 1989). Is it fair to conclude that three independent voices are an upper limit of texture complexity that listeners track?

This question relates to whether timbre as a grouping/segregation cue affects any upper limit of complexity. The *timbral differentiation* principle (Huron, 2016) states that timbrally heterogeneous mixtures are easier to segregate compared to homogeneous mixtures. Siedenburg et al. (2021) put this principle to test with excerpts from JS Bach’s *The Art of the Fugue*. Five-second excerpts with homogeneous or heterogeneous instrumentation of 2–4 musical voices were presented from spatially separated loudspeakers and preceded by a short cue for signaling the target voice. Listeners tracked the cued voice and detected whether an amplitude modulation was imposed on the cued voice or a distractor voice. Performance was generally better in conditions with fewer voices, although there was an interaction between the number of voices and the instrumentation: performance degraded less drastically with an increase in the number of voices for timbrally heterogeneous mixtures compared to homogeneous mixtures. That is, timbral differentiation counteracts the limited density principle, quantified by the number of simultaneously active voices.

A case where sound complexity is clearly audible and yet not necessarily daunting is that of sound mass composition. As noted by McAdams (1984) “Listen to the large sound masses played by the strings in Ligeti’s Atmosphere or Penderecki’s Threnody to the Victims of Hiroshima and ask yourself how many individual instruments are playing simultaneously in that section. Certainly you can identify that there are ‘many’, but ‘how many’ is difficult to determine because the sounds are all so closely related that they obscure one another and are not individually distinguishable.”(p. 18) Douglas et al. (2016) studied the perception of Ligeti’s *Continuum*, a piece with a rapid stream of eighth-note dyads. Listeners heard one continuous texture at ca. 20 attacks per second, but a variety of factors (rhythm, timbre, register) affected this situation. Noble and McAdams (2020) proposed to define sound mass as “a perceptually homogeneous and dense auditory unit integrating multiple sound events or components while retaining an impression of multiplicity. Although their acoustical correlates may be highly complex, sound masses are perceptually simple because they resist perceptual segregation in one or more parameters (e.g., pitch, rhythm, timbre).” (p. 239) This definition nicely differentiates between acoustic (source) complexity and the perceptual complexity in the minds of listeners. The authors argued that sound mass often exploits MSA principles in reverse, fostering attentional focus on the musical totality (the mass) by exceeding or subverting the perceptual independence of its individual parts.

## Individual differences in musical scene analysis

MSA involves parsing complex auditory signals into discrete, recognizable streams. This ability, however, is not uniform across individuals. For some, the ability to identify a single instrument amidst the aggregated layers of an orchestral performance seems almost intuitive (i.e., conductors), while others struggle to discern even the most prominent instruments in rather simple musical arrangements. This disparity illustrates that MSA is partially determined by individual differences, which are rooted in determinants of peripheral auditory processing such as individuals’ hearing sensitivity, and determinants of cognitive processing such as attentional control and memory. Understanding these individual differences is crucial for developing a comprehensive picture of MSA.

### Peripheral determinants

Damage along the auditory pathway can substantially compromise auditory perception, affecting frequency resolution, temporal processing, and spatial hearing (Moore, 2007). Such degradations directly limit the availability and reliability of the acoustic information that supports MSA, including pitch, timbre, harmonicity, and temporal cues. As a consequence, listeners with hearing impairment often experience pronounced difficulties in forming and maintaining distinct auditory streams within musical scenes. These challenges are vividly illustrated in experimental contexts. Siedenburg et al. (2020) had listeners discriminate melodies and timbres in the presence of a musical accompaniment and in a staircase procedure that adaptively changed the signal-to-masker ratio. It was observed that older hearing-impaired listeners required substantially higher signal-to-masker ratios – on the order of 10 dB – compared to younger normal-hearing listeners to discriminate target signals. Importantly, these differences arose with stimuli presented at medium loudness levels, indicating that reduced audibility alone cannot account for the observed deficits. Extending these findings to ecologically valid musical material, Siedenburg et al. (2021) demonstrated that older adults with hearing loss showed poorer performance in tracking individual voices, even after being compensated by hearing aids.

Using an instrument detection task based on real-world, multi-track recordings from different musical genres (including pop, rock, EDM, etc.), Hake et al. (2023) showed a systematic decline of performance with increasing hearing loss severity among a large and heterogeneous online sample. Whereas listeners with mild hearing loss showed moderate reductions in accuracy, individuals with severe-to-profound hearing loss frequently performed at or near chance level. This pattern has been replicated across independent samples. For example, using the same paradigm, Benjamin and Siedenburg (2024) reported that younger normal-hearing listeners consistently outperformed older hearing-impaired listeners across all target categories, irrespective of spectral manipulations applied to the musical mixes, indicating substantial limitations in the segregation of concurrent musical streams. Hake et al. (2025) observed that hearing aids compensate peripheral deficits to some extent, with fast-acting hearing-aid compression generally scoring higher compared to slow-acting compression (even though the sound quality of fast-acting compression was rated more poorly).

Importantly, a common limitation of many of these studies is that they contrast older hearing-impaired listeners with younger normal-hearing controls, thereby conflating the effects of hearing loss with those of aging. The latter is associated with both peripheral auditory decline and central neurocognitive changes, which jointly affect auditory perception and scene analysis in complex and interconnected ways (Moore, 2015). Progressive cochlear damage, often related to cumulative noise exposure, is thought to lead to degraded auditory input and structural changes along the auditory pathway, including synaptic loss, auditory nerve fiber degeneration, and reduced neural synchrony (Bramhall et al., 2019; Kujawa & Liberman, 2009).

In summary, hearing sensitivity and impairments play a crucial role in shaping individual differences in MSA. Yet, even among those with normal hearing, significant performance variability persists. This suggests that there exist other factors that are critical contributors to individual differences in MSA.

### Cognitive determinants

Although bottom-up auditory constraints play a crucial role in shaping MSA performance, they do not fully account for the pronounced individual differences observed among listeners with similar hearing thresholds (Hake et al., 2023; Siedenburg et al., 2020). This variability points to the importance of top-down cognitive and experiential factors that influence how musical scenes are perceived.

Early neuroimaging work already highlighted the role of listener-dependent effort in the context of MSA. Janata et al. (2002) asked participants to attend to individual instruments within polyphonic musical excerpts selectively and to rate the perceived difficulty of the task. Subjective difficulty ratings varied markedly across listeners, with some participants finding the task effortless (2–3 on a seven-point Likert scale), while others rated it as highly challenging (5–6) – despite comparable hearing status and musical background. Conditions perceived as more demanding were associated with increased recruitment of attentional networks and regions implicated in working memory, suggesting that individual differences in MSA are reflected not only in subjective evaluation but also in the cognitive resources engaged during listening. These findings indicate that, even under identical acoustic conditions, MSA can place substantially different cognitive demands on listeners.

Musical training represents a particularly potent and systematic source of such top-down influences on MSA. Through prolonged exposure to complex polyphonic textures, musicians acquire refined representations of musical structure and potentially develop enhanced control over attentional selection within musical scenes as a consequence (e.g., Disbergen et al., 2018). Consistent with this view, behavioral studies consistently show that musicians outperform non-musicians in tasks requiring the segregation of melodies from competing musical material. For example, Coffey et al. (2019) showed that musicians are better able to segregate and identify melodic patterns embedded in musical masking noise than non-musicians, with this advantage remaining stable across a wide range of signal-to-noise ratios. More recent work replicated this effect and further demonstrated that this enhanced segregation ability is closely linked to listeners’ auditory working memory capacities, which largely mediate the association between musical training and performance in complex musical scenes (Liu et al., 2025), further substantiating the interplay of higher-order cognitive functioning and MSA performance. Wenhart et al. (2019) showed that, among a group of professional musicians, individuals with absolute pitch outperform those with relative pitch in melody segregation tasks. These findings (once more) argue against the simple dichotomy of musicians vs. non-musicians and instead support a multifaceted account of musical expertise and skills.

However, the relationship between musical training and MSA is not uniformly observed across different samples. For example, despite using the same paradigm, Bürgel et al. (2021) did not find a significant association between a continuous index of musical training and target detection performance, whereas Hake et al. (2023) reported a reliable positive relationship in a larger and more heterogeneous sample. Extending this work, Hake et al. (2025) assessed musical training alongside age, working memory capacity, and hearing loss severity, and demonstrated that the association between musical training and MSA performance remained robust after accounting for these factors. Follow-up analyses further indicated that the magnitude of this training effect had similar effect sizes compared to that of mild-to-moderate hearing loss, underscoring the behavioral relevance of musical training for MSA.

Beyond quantifying the effects of musical training on ASA ability, one may study qualitative shifts in auditory organization. Schneider et al. (2005) documented inter-individual differences in pitch shift perception. They presented shifts with the gross spectral envelopes of harmonic tone complexes going in the opposite direction of the fundamental frequency (f0). These were artificial stimuli, constructed through incomplete selection of partial tones for each pair. Here, the direction of the spectral envelope shift dominated perception of some listeners, whereas f0 dominated shift perception for others. Siedenburg et al. (2023) also observed individual differences in frequency shift perception, albeit only for a much more broad set of stimuli. This type of research may be related to the Helmholtzian notion of analytic (spectral) vs. synthetic (f0-based) listening. It seems reasonable to state that musicians are capable of perceiving more detail in music due to an analytical mode of listening compared to non-musicians. This assumption is supported by the review of selective listening abilities and the effect of musical training above. However, one may also argue that, in trained individuals, there exists a bias toward local vs. global features in the music. In a series of studies, Susini and colleagues have recently demonstrated such a bias (Bouvier et al., 2025; Susini et al., 2020, 2023). They played listeners carefully constructed melodies that were varied in terms of their local or global contour (pattern of up and down movements), or both. Listeners were instructed to attend to either local or global characteristics of the melody and report when they heard changes in the melody. Musicians generally achieved better scores. Interestingly, non-musicians showed a bias towards global features, whereas musicians were biased to attend to local features. Musical training over longer time spans thus appears to contribute to a perceptual reorganization that diminishes the dominance of global features of melodies in favor of local features. It seems plausible that this pattern may extend towards ASA in natural music, where musicians may be biased to analytically segregate chords or textures into their individual components and non-musicians may be biased to attend to global properties of the music, even though we are not aware of any empirical work that has documented such effects.

Recent work has extended the notion of individual differences to considerations of cultural background, which plays a critical role in the formation of musical knowledge and resulting schema-based segregation. In a study comparing fusion ratings of sound pairs, McPherson et al. (2020) compared a group of US Western listeners with Amazonian listeners with little exposure to Western music. Both groups were more likely to perceive note combinations related by simple integer ratios as a single, fused sound. This suggests that acoustic constraints on sound segregation impose perceptual structure on combinations of notes, even for listeners with minimal exposure to Western harmony. However, fusion did not predict aesthetic judgments of intervals in either Western listeners or Amazonians, the latter showing indifference to consonance and dissonance. These findings point to universal perceptual mechanisms that may underlie cross-cultural regularities in musical systems, while also indicating that such mechanisms interact with culture-specific factors to give rise to musical phenomena such as consonance.

## Perspectives

The processes of MSA reviewed above contribute to structuring music in the minds of the listeners. But if hearing needs to separate target signals from noise and if this imposes processing costs for the auditory system, why then is music itself such a rich signal, stuffed with auditory events and streams, often without clearly defined target signals? Why are these busy, multi-source music signals such a pleasure to listen to? These questions lead us to address the relationship between ASA principles, musical structure, and pleasure.

### Music is attuned to ASA

Music composed according to the rules of Baroque counterpoint often serves as a quintessential example of polyphonic music. The resulting textures contrast with the musical scenes in figure-ground textures, where a main melody is set “in front of” a background accompaniment. In Baroque polyphony, individual voices move seemingly independently, and listeners may switch attention from one voice to another. The compositional regularities underlying polyphonic music from the Baroque period are commonly referred to as voice-leading rules. These rules traditionally play an important role in Western music pedagogy, providing guidelines on how to construct polyphonic textures, in which musical voices or lines move independently. Huron (2016) described that “the goal of voice leading is to facilitate the listener’s mental construction of coherent auditory scenes when listening to music” (p. 88). Wright and Bregman (1987) and Huron (2001) first outlined how music theory rules of counterpoint correspond to ASA principles. When composers aim for textures with independent musical voices, the principles of ASA will guide the relationship of these voices. Notably, effective voice-leading requires clear auditory stream integration within each of the individual parts and clear auditory stream segregation between each of the concurrent parts. These basic assumptions then lead to central voice-leading rules (Huron, 2016). For example, *Move the shortest distance possible*: Moving the shortest distance possible will avoid large leaps, keeping voices in their place and stabilizing integration of each part. *Avoid voice crossings*: Voice crossing would lead to ambiguous continuation of voices, thus they should be avoided. *Avoid parallel octaves and fifths*: Octaves and fifths fuse very strongly; thus lines moving in parallel with these interval relations could yield fusion of multiple parts into one line. *Limited density*: Limited density of textures reacts on the limited cognitive capacity of listeners; thus polyphonic textures supposedly exhibit a sweet spot of three independent lines that is somewhat independent of the number of individual instruments/sections in the musical ensemble. Composers follow rules of voice leading to reach perceptual clarity and ensure independence of voices.

McAdams et al. (2022) have outlined a complementary, perceptually grounded taxonomy of musical orchestration. They define orchestration as the “selection, combination, and juxtaposition of instruments at different pitches and dynamics to achieve a particular sonic goal. This definition could be extended from ‘instruments’ to ‘sounds’ more generally to encompass the voice, as well as recorded and electroacoustic sound sources.” (p. 1) In concurrent grouping, composers may aim for different types of blends: augmentation, emergence, heterogeneity/non-blend. In sequential grouping, composers may target for (time-variant) stream integration, segregation, or the stratification of orchestral layers. In segmental grouping, composers may create timbral contrasts, shifts, echoes, antiphonal contrast/call and response, timbral juxtapositions, and sectional boundaries. These categories can act as part of a hierarchical organization: blended sounds can form a stream that lives within a foreground layer that is itself located within a section defined by a boundary (McAdams et al., 2022). Generally, multi-source musical scenes afford increased differentiation of musical structure within the music and refined communication of affective intentions.

These considerations suggest that music composition on a note level (e.g., by following the rules of counterpoint) or on an instrument level (by using orchestration techniques) is closely attuned to ASA. A similar case can be made beyond (classical) music composition, where music producers work hard to find the right balance between simplicity and complexity of the musical scenes they produce, and sound engineers work hard to create perceptual transparency and give instruments their space, which may manifest in either clear or muddy listening experiences (even though we are not aware of research on this topic in this domain). Music performers strive to sync their musical phrasing, intonation, tone or sound, and timing with fellow ensemble members to collectively sculpt one musical idea. Although not necessarily known as a music critic, Bregman (2015) notes in this regard, “the genius of the great Canadian pianist, Glen Gould, was that he made polyphony easier to decipher. His recordings may make it accessible to a wider audience” (p. 14). Room acoustics shape musical clarity, and performers react to the acoustics of a performance space (Schärer Kalkandjiev & Weinzierl, 2013). In these domains of musical practice, musicians face their interactions with musical sound being constrained by the principles of MSA. By means of analogy, one may argue that MSA acts as a type of “invisible hand” of music generation, which regulates individual actors (composers, performers, sound producers), without individuals being necessarily aware of the underlying perceptual processes at play (cf., Siedenburg, 2025).

### Aesthetic affordances of multi-source music

Composers might want to aim for perceptual clarity by creating affordances for stream segregation within musical textures. Yet, music remains an artifact, and there is no principled need to strive for clarity. Indeed, Bregman (1990) considers music as a fundamentally chimeric stimulus:

“The Chimera was a beast in Greek mythology with the head of a lion, the body of a goat, and the tail of a serpent. We use the word chimera metaphorically to refer to an image derived as a composition of other images. An example of an auditory chimera would be a heard sentence that was created by the accidental composition of the voices of two persons who just happened to be speaking at the same time. Natural hearing tries to avoid chimeric percepts, but music often tries to create them. It may want the listener to accept the simultaneous roll of the drum, clash of the cymbal, and brief pulse of noise from the woodwinds as a single coherent event with its own striking emergent properties. The sound is chimeric in the sense that it does not belong to any single environmental object.” (p. 459)

Accordingly, decoding multivalent stimuli and appreciating ambiguity is a key part of music perception. A very basic case in point is the chord, which comprises individual tones that may be heard separately, yet form a perceptual unity with emergent qualities. Studying ambiguity empirically, Pelofi et al. (2017) showed that participants with musical training exhibited less confidence in processing physically ambiguous stimuli compared to participants without formal musical training. Their participants judged pitch shifts of Shepard tones as going up or down and also provided confidence ratings for these judgments. Interestingly, the confidence of non-musicians peaked at the maximally ambiguous half-octave shift, but musicians appeared to be more aware of this ambiguity, yielding a valley in confidence and longer response times. It is not directly clear what this finding implies for natural musical scenes, where ambiguity arises on multiple levels of the music, both vertically and horizontally. Similarly, McAdams et al. (2025) found that musicians reported higher perceived multiplicity in orchestral music than non-musicians, even for excerpts that blended fairly well, reflecting greater awareness of the multiple affordances of musical structure. Yet, the “sense” of multiplicity without a cognitive awareness of the number of sources could arguably be an important part of the magic of texture in music. Polyphonic musical textures encompass unity and diversity, and because the same acoustic surface may admit multiple plausible interpretations, perceptual grouping can shift with attention, expertise, or listening goals. Analytical interpretations may even vary among trained musicians. From a theoretical standpoint, MSA should consequently be framed as probabilistic inference rather than deterministic decomposition.

More fundamentally, one may ask why musical stimuli should be clearly structured or intelligible in the first place, given the aesthetic appeal of ambiguity. According to Huron, the aesthetic appeal of polyphonic music lies in its ability to act as “ear teasers”, a way of offering a perceptual riddle to the ear that provides pleasure, once solved successfully. It remains to be determined how “success” would be defined exactly here, but correctly segregating physically separate sound sources could be part of it. In his own words, “an ear teaser is any complex musical texture or acoustic scene that nevertheless affords clear scene analysis parsing with a consequent pleasurable effect for listeners who successfully resolve the sensory challenge.” (Huron, 2016, p. 198). According to this account, biological systems are geared towards successful scene parsing regardless of perceptual modality, and any failure in scene parsing reduces an organism’s biological fitness. In turn, biologically advantageous behaviors are assumed to evoke positive feelings. Huron speculates that the amount of pleasure evoked by “successful” scene analysis then is proportional to the perceptual difficulty posed by the scene.

As an illustration of this idea, Huron takes the example of reverberation. Reverberation is the sum of all sound reflections in rooms and a very important device in music production. From an ASA perspective, however, reverberation is often thought to be detrimental to stream segregation because it smears the temporal waveform. Huron suggests (without presenting empirical evidence) that reverberation adds to the complexity of a musical scene and makes scene parsing more challenging. Because it creates a perceptual challenge, reverberation is a preferred property (or *clothing*) of sound. Reverberation has immediate sensory and affective impact but does not fulfill any musical function from a structural point of view. However, comparing MSA abilities in conditions with and without reverb, studies have observed only small, if not marginal, effects on scene transparency (Jacobsen et al., 2026). Thus, empirical data suggest that its role as an ear teaser seems limited. Yet, the role of reverberation as a device in music production is strong. Reverberation envelops listeners, giving them the feeling of being wrapped in sound, instead of being separated from the sound source (as in an anechoic chamber in the most extreme case). One may even speculate that most listeners prefer unreverberated speech but reverberated music, especially so for a small number of instruments. Why then is reverb such a critical part of musical sound, when it does not convey musical structure? And back to the general question, why does polyphonic music have such a great effect on listeners? There is certainly much to be explored in this realm in the intersections of MSA and empirical aesthetics.

Suffice it to say that beyond structural differentiation and ear teasing, multi-source textures create rich affordances for envelopment and “being in the sound” (Solomos, 2019). We speculate that the resulting perceptual complexity enables sensory immersion and transports listeners to a different perceptual place, where objects and events do not follow the laws of physics, but the principles of MSA. Composers can sculpt these worlds, with the potential for altered perception of time (Noble, 2018). As argued by Touizrar (2026), multi-source music imputes the experience of spatiality, and therefore centers the listener in the vantage of a central character who is surrounded by events that spatialize on a phenomenological level. The clarity of the scene, which can be regulated with MSA principles, then may serve as the basis for how readily listeners can orient in experienced musical space.

## Conclusion

This review has surveyed musical scene analysis (MSA) as a multifaceted process shaped by interactions between acoustic structure, perceptual organization, cognitive expectations, and cultural experience. Building on foundational work in auditory scene analysis (ASA), we traced how Gestalt principles and primitive grouping cues such as common onset, harmonicity, and co-modulation provide bottom-up constraints on the parsing of musical sound. At the same time, schema-based processes and learned regularities play a crucial role in guiding grouping and segregation, highlighting the importance of top-down influences in musically meaningful contexts. Across experimental paradigms, minimalist designs have proven essential for isolating core perceptual mechanisms, while ecological approaches reveal how these mechanisms operate in complex, real-world musical scenes. The review of stimulus-based aspects underscores the fact that blend, salience, and complexity are important determinants of MSA. Likewise, listener factors ranging from peripheral auditory limits to higher-level cognitive and cultural knowledge critically shape how musical scenes are experienced. Finally, the theoretical perspectives discussed suggest a reciprocal relationship between music and MSA: musical practices appear attuned to the capacities and constraints of ASA and multi-source music exploits and at times overwhelms these processes to afford aesthetic richness and immersion. Together, these insights position MSA as central to understanding not only how listeners organize sound, but also how musical structure and experience emerge from perceptual organization.

There are many outstanding questions to be addressed by future research on MSA. These concern a more detailed characterization of the concrete psychoacoustic processes that underlie the tracking of multiple voices in polyphonic music. Another important avenue for future research concerns individual preferences in MSA: could there be clusters of listeners who prefer compact and fused musical scenes over clearly segregated sources in a mix, and can we observe a relationship to determinants such as hearing loss or age? How can hearing aid technology be improved to provide a clear perception of musical scenes? Yet another area that we have not touched on at all concerns multisensory integration, where future work may reveal how vision and/or vibrotactile perception co-shape the experience of musical scenes.

## Data Availability

Links to (interactive) sound examples can be found here: https://uol.de/en/music-perception/sound-examples/msa-review

## Appendix: Glossary

**Table:**

Term	Definition
Blend	The perceptual merging of multiple sounds or elements into a single, unified auditory event rather than separate parts.
Common fate	A grouping principle where sound elements changing together over time are heard as belonging to the same source.
Common onset	A grouping principle where sounds that begin at the same time are heard as coming from the same source or origin.
Event	A perceptual unit perceived as a distinct occurrence. Sounds from multiple sources can fuse as part of one event.
Fusion (integration)	The process by which sounds from separate sources are perceived as a single sound rather than multiple independent elements.
Gestalt	A notion emphasizing that perception organizes sensory input into meaningful wholes, not merely collections of parts.
Harmonicity	The degree to which frequency components align as integer multiples of a fundamental frequency, promoting perceptual fusion.
High voice superiority	The effect by which the highest voice in polyphonic music stands out perceptually.
Pitch	The perceived height of a fused sound event, primarily related to its fundamental frequency, allowing ordering from low to high.
Primitive grouping	Automatic, low-level perceptual grouping based on basic sensory cues, occurring without conscious attention or learning.
Schema	A piece of general perceptual knowledge that shapes audition by providing expectations about how sounds or events typically unfold or relate.
Segment	A perceptually distinct portion of a sound stream, separated from others by changes in acoustic or temporal features.
Segregation (fission)	The perceptual separation of sounds into distinct perceptual streams.
Sound mass	When multiple sounds are integrated into one “thick” texture.
Stream	A perceptual representation of a sound sequence, formed by grouping related auditory events across time.
Timbre	The quality of a fused sound event that qualifies it beyond pitch and loudness.
Vocal salience	The process by which human vocal sounds tend to capture auditory attention more readily than other instruments in musical mixtures.

## Footnotes

1 https://uol.de/en/music-perception/sound-examples/msa-review

2 https://classicalexburns.com/2022/05/23/gyorgy-ligeti-lontano-in-the-distance/

3 Online, they can be accessed via https://www.themusiclab.org/bregman.
